# Post-traumatic stress disorder: the role of the amygdala and potential therapeutic interventions – a review

**DOI:** 10.3389/fpsyt.2024.1356563

**Published:** 2024-06-06

**Authors:** Lori L. Davis, Mark B. Hamner

**Affiliations:** ^1^ Mental Health Service, Birmingham VA Health Care System, Birmingham, AL, United States; ^2^ Department of Psychiatry, University of Alabama at Birmingham Heersink School of Medicine, Birmingham, AL, United States; ^3^ Department of Psychiatry, University of Alabama College of Community Health Science, Tuscaloosa, AL, United States; ^4^ Department of Veterans Affairs, Ralph H. Johnson VA Medical Center, Charleston, SC, United States; ^5^ Department of Psychiatry and Behavioral Sciences, Medical University of South Carolina, Charleston, SC, United States

**Keywords:** post-traumatic stress disorder, PTSD, amygdala, pathophysiology, pharmacotherapy, transient receptor potential canonical ion channel

## Abstract

**Introduction:**

Post-traumatic stress disorder (PTSD) is a psychiatric disorder triggered by exposure to a life-threatening or sexually violent traumatic event, and is characterized by symptoms involving intrusive re-experiencing, persistent avoidance of associated stimuli, emotional and cognitive disturbances, and hyperarousal for long periods after the trauma has occurred. These debilitating symptoms induce occupational and social impairments that contribute to a significant clinical burden for PTSD patients, and substantial socioeconomic costs, reaching approximately $20,000 dollars per individual with PTSD each year in the US. Despite increased translational research focus in the field of PTSD, the development of novel, effective pharmacotherapies for its treatment remains an important unmet clinical need.

**Observations:**

In this review, we summarize the evidence implicating dysfunctional activity of the amygdala in the pathophysiology of PTSD. We identify the transient receptor potential canonical (TRPC) ion channels as promising drug targets given their distribution in the amygdala, and evidence from animal studies demonstrating their role in fear response modulation. We discuss the evidence-based pharmacotherapy and psychotherapy treatment approaches for PTSD.

**Discussion:**

In view of the prevalence and economic burden associated with PTSD, further investigation is warranted into novel treatment approaches based on our knowledge of the involvement of brain circuitry and the role of the amygdala in PTSD, as well as the potential added value of combined pharmacotherapy and psychotherapy to better manage PTSD symptoms.

## 1 Introduction

Post-traumatic stress disorder (PTSD) is a psychiatric condition characterized by a history of exposure to a life-threatening or sexually traumatic event leading to re-experiencing, avoidance, negative thoughts, and hyperarousal symptoms resulting in occupational and social function impairments (1). The development of novel and efficacious pharmacotherapies for this disorder represents an important unmet clinical need, as no new drugs have been approved by the US Food and Drug Administration (FDA) for PTSD treatment since sertraline (1999) and paroxetine (2000) (2–4). Approximately 58% of PTSD clinical trial participants still meet diagnostic criteria following cognitive behavioral therapy and only 32%–66% achieve a sufficient level of functioning (5, 6). In a meta-analysis of 66 randomized controlled trials assessing PTSD medication efficacy, 42% of SSRI-treated patients did not demonstrate symptom improvement, and varying efficacy and tolerability of current medications suggests further research is warranted (7). Despite remarkable progress in our understanding of the neurobiology of PTSD over the past 15 years (8), further investigation of the biological correlates and pathological mechanisms of PTSD symptoms are required to enhance diagnosis and treatment (9). Thus, considering PTSD prevalence and the significant economic and clinical burden associated with the disorder at a societal and individual level, uncovering the psychosocial and biological correlates of PTSD is essential in assessing the current treatment landscape and facilitating emerging therapy development.

## 2 Observations

### 2.1 PTSD

Diagnosis of PTSD is based on exposure to actual or threatened death, serious injury, or sexual violence, according to the Diagnostic and Statistical Manual of Mental Disorders 5^th^ Edition (DSM-5) (1). Traumatic exposures can vary and confer diverse levels of risk for PTSD depending on their nature and severity (10). Individuals with PTSD experience symptoms in 4 domains: intrusive re-experiencing, persistent avoidance behaviors, emotional and cognitive disturbances, and hyperarousal and reactivity, including difficulty sleeping and concentrating (1, 4, 11). Although remission can occur within months of onset in some cases, symptoms typically last for many years (10).

A systematic literature review of PTSD prevalence data in the United States from 2015 to 2019 reported that the 1-year prevalence of PTSD among the civilian population ranged from 2.3% to 9.1%, with a lifetime prevalence of 3.4%–26.9%, while for the military/veteran population, rates were higher at 6.7%–50.2% and 7.7%–17.0%, respectively (12). The large prevalence variation in military groups was attributed to heterogeneity in study design and study population (12). Despite increased prevalence, the military/veteran population only accounts for a small proportion of the PTSD population. There are double the number of men and 25 times the number of women with PTSD in the civilian population by comparison, highlighting that PTSD is not exclusive to veterans/military service members (13). Women in the US tend to have higher lifetime PTSD prevalence rates than men (8%–13% for women, 4%–6% for men), and 2018 estimates calculated that women accounted for 66.4% of the overall PTSD population in the US (12, 13). The societal impact of PTSD in 2018 was calculated to have an economic burden of $232.2 billion in the US, with costs of ~$20,000 per individual living with PTSD (13). A quality assessment of recent economic burden and cost-of-illness in PTSD studies reported that cost varied according to the type and severity of the associated trauma; sexual assault in women was associated with a high economic burden (14). As PTSD is underdiagnosed and undertreated (12, 13), its true economic burden likely exceeds these estimates.

#### 2.1.1 Neurobiology of PTSD

Although up to 84% of the general population will experience a traumatic event (15), only a fraction develop PTSD, indicating that PTSD susceptibility varies depending on risk and resiliency factors including the nature and severity of the aversive event, sex, inter-individual differences in coping styles and genetics, and the neurodevelopmental period in which the trauma was experienced (16–20). The specific biological and environmental factors that determine susceptibility and resilience to PTSD pathology remain unclear (9), necessitating better understanding of the neurobiological processes that contribute to PTSD pathobiology.

#### 2.1.2 The role of the amygdala

The amygdala complex is situated in the temporal lobe of the brain and comprises several subcortical nuclei (21–23). It is has 2 main regions: the primitive centrocorticomedial complex and the basolateral complex (BLA) (21, 23). Functions of the amygdala include encoding fearful stimuli, allowing the brain to direct behavioral responses upon encountering such stimuli, as well as playing a role in fear generalization, arousal, and reward processing; thus, it has been widely implicated in PTSD pathophysiology (21, 24, 25).

The amygdala controls both innate fear, and acquired/learned fear, with the latter linked to escape or avoidance behaviors to previous noxious stimuli (22) which are associated with functional changes in the BLA (26). Considering the important role of these amygdala nuclei in fear conditioning, it follows that morphological and functional changes in these regions may explain the reinforcement of memories related to traumatic events in PTSD (27). Reports indicate that morphological and functional changes in the amygdala are evident, resulting in disrupted control of fear generalization, arousal, and reward processing in individuals with PTSD (21, 24, 25, 28, 29). Specifically, decreases in amygdala nuclei volumes have been reported to be associated with symptoms of PTSD (21, 24, 29). Reduced grey matter volume in limbic brain regions including the amygdala are correlated with sleep disturbances (insomnia and nightmares) in patients with PTSD (30), which may contribute to symptom severity (31, 32). Analysis of links between BLA and centrocorticomedial complex volumes with PTSD symptom severity in 47 young survivors from the 2011 Norwegian terror attack revealed an inverse relationship between symptom severity and amygdala nuclei volume 24–36 months post-trauma (21), supporting associations between these factors and PTSD. The conflicting reports of increased amygdala activity and reduced volumetric data is consistent with previous findings in PTSD (33), and may be partly explained by a lack of distinction between regional changes in specific amygdala nuclei and those of the whole amygdala (21). A recent scoping review of structural neuroimaging studies examining hippocampus and amygdala subregions in adults with PTSD reported that only 5 of the 21 eligible studies focused on volume changes in the amygdala, highlighting the anatomical complexity and methodological challenges of establishing structural changes in this brain region (34). Functional neuroimaging, alternatively, may hold greater promise in understanding the role of the amygdala in PTSD pathophysiology (35). Abnormal functional connectivity between the amygdala and other parts of the brain has been demonstrated in PTSD, with stronger connectivity between subregions of the amygdala, particularly the BLA, spanning to the prefrontal cortex (PFC) compared with healthy controls (25, 28, 35). The amygdala communicates the salience of the threat cues to the PFC, which in turn regulates emotional and behavioral fear responses to provide top-down emotional control (36). It is not surprising, therefore, that such connectivity is altered in a condition associated with enhanced fear responses. Furthermore, white matter changes in the ventromedial PFC that negatively correlated with symptom severity have been demonstrated in individuals with PTSD within 2 days of experiencing traffic-accident-induced trauma (37). Further support for BLA involvement in the regulation of fear memories and responses is provided by animal studies demonstrating that BLA neurons trigger hippocampal neurogenesis in fearful contexts (38), and also play an essential role in reinstating fear response to a previously extinguished fear memory (39); patients with PTSD often have spontaneous and regular traumatic memory retrieval (40).

PTSD symptoms develop not only from associative learning such as fear conditioning, where trauma-related stimuli trigger a fear response, but also from non-associative learning processes such as habituation or sensitization to aversive cues (27). These non-associative processes involve incremental escalations of defensive fear responses to aversive or harmful stimuli that can be responsible for pain (41). Individuals with PTSD are believed to be more sensitive to stimuli, to over-generalize fearful stimuli or events, and are unable to extinguish learned or conditioned fear (11). Sensitization to neutral stimuli is evident in patients with PTSD, for example, where the traumatic experience becomes associated with a previously neutral stimulus that has now acquired a threatening meaning (42).

Inflammatory biomarkers including cytokines and C-reactive protein have shown to positively correlate with anxiety symptoms and negatively correlate to functional connectivity between the lower amygdala and ventromedial PFC in women with anxiety and PTSD (43). This association between functional changes in amygdala-PFC connectivity and stressor-induced inflammation is also evident in healthy females that demonstrated greater neural activity in the left amygdala in response to negative social feedback that correlated with greater changes in IL-6 (44). Given that elevated serum levels of pro-inflammatory cytokines are commonly reported in individuals with PTSD (45), and that cytokines can cross the blood-brain barrier (46, 47), it is possible that trauma-induced inflammation may contribute to pathophysiological changes in stress-related brain areas such as the amygdala (48). These findings are well supported by clinical studies, which have shown an association between inflammation and PTSD symptoms (45). Animal studies have also shown that heightened inflammation impairs extinction of fear memory (49, 50), and administration of the interleukin-6 (IL-6) cytokines to the BLA prior to extinction training impairs fear extinction memory (51), suggesting inflammation can alter amygdala fear regulation.

Central to the brain circuitry underlying fear memory processing in disorders such as PTSD is the lateral amygdala, which is the input for stimuli during fear conditioning in animals (11, 22, 52, 53). Therefore, impairment of fear or extinction responses in individuals with PTSD may be indicative of diminished synaptic plasticity, particularly in pathways associated with the lateral amygdala where sensory input is initially processed (22, 52, 54, 55). This early synaptic plasticity in response to fear stimuli in the lateral amygdala is essential in signaling the presence of danger to other connected regions such as the PFC, hippocampus, and hypothalamus so that defensive behavioral and physiological stress responses can be readied for the fight/flight reaction (27, 56). Overall, the role of the amygdala in fear responses, and the dysfunctional activity and connectivity of this brain region in the fear conditioning response and impaired fear extinction in PTSD, is strongly supported (27, 36, 56).

#### 2.1.3 Transient receptor potential canonical ion channel subfamily C in the CNS

To date, 7 types of transient receptor potential canonical (TRPC) ion channels have been identified (57, 58). TRPC channels are distributed in several brain areas, particularly the amygdala and hippocampus (22, 59), and are reported to be moderately expressed in rodent PFCs (60). TRPC channel activation leads to an influx of Na^+^ and Ca^2+^ ions causing both membrane depolarization and elevation of intracellular Ca^2+^, impacting significantly on cellular function and intracellular signaling (58). The diverse structural properties and distribution of the various TRPC channel types determine the different functions associated with their activation, but they can be broadly described as cellular sensors, due to their regulation of intracellular calcium (58). As they have diverse biological roles, TRPCs are considered potential drug targets for a broad range of disorders such as respiratory disorders, diabetes, cancer, as well as neurological and psychiatric diseases (61).

In the limbic regions, TRPC1, TRPC4, and TRPC5 are the most common, primarily within hippocampal pyramidal cells and the amygdala (62–64). TRPC4 and TRPC5 are homologous proteins that form ion channels involved in the regulation of neuronal growth, axon guidance, synaptic plasticity, and cellular excitability (60). Impairments in these cellular processes contribute to important brain functions, including working memory, hippocampal dysfunctions such as seizures, and fear responses (62, 64, 65). TRPC knockout mice (TRPC5−/−) exhibited diminished fear-related behaviors, indicating that TRPC5 plays a key role in fear responses in mice (22). A similar study in TRPC4−/− mice demonstrated diminished anxiety or innate fear relative to wild-type mice (59). Interestingly, these anxiolytic-like behaviors in TRPC4−/− mice were only present in stressful test conditions (bright versus dim lighting) suggesting the TRPC4 effects are more readily revealed in anxiety-provoking situations (59), a desirable effect in the treatment of PTSD. However, no differences in learned or conditioned fear responses were observed in these knockouts (59). A link between TRPC subtypes and neuroinflammation is also evident from animal studies. Transgenic mice with IL-10 deficiency in microglial cells that express elevated levels of pro-inflammatory cytokines also exhibited decreased TRPC4 and TRPC5 in the hippocampus, and decreased TRPC5 in the PFC and amygdala when compared to wild-type control (66, 67), suggesting a link between TRPC and neuroinflammation in stress-related brain areas. Considering the evidence implicating amygdala involvement in PTSD symptoms (24), the location of both TRPC4 and TRPC5 in the amygdala (22, 59, 60) and their link with local neuroinflammation (66, 67), in addition to the reduced fear and anxiety demonstrated in knockout mice (TRPC4−/− rodents and TRPC5−/− rodents) (22, 59), it is reasonable to hypothesize that dampening amygdala activation of the fear response by blocking these ion channels may reduce PTSD symptom severity.

### 2.2 Current treatment strategies

#### 2.2.1 Evidence-based approach to care

Currently, both psychological and pharmacologic strategies are employed for PTSD management (1). Recommended forms of PTSD treatment include psychological strategies, shared decision-making, collaborative care, and trauma-focused therapy; both shared decision-making and collaborative care involve reviewing the relevant diagnosis, available treatment options, and use of decision aids to improve clinical outcomes (1). Manualized, trauma-focused therapies such as prolonged exposure therapy, eye movement desensitization and restructuring, cognitive processing therapy, and cognitive behavioral therapy have also been shown to reduce PTSD symptoms (1). Cognitive behavioral therapy is also effective in alleviating PTSD-associated insomnia (68). The efficacy of psychotherapies is strongly supported by current literature; however, access is often limited (69). Since the COVID-19 pandemic, internet-based delivery of treatments has increased and offers potential avenues to widen access and reduce costs (70). Challenges identified in clinical studies examining efficacy of these therapies include issues with engagement and early drop-out rates (71), which may be improved by facilitating access to technology-based delivery or with combined efficacious pharmacotherapy (70, 72). In recent years, non-invasive brain stimulation techniques, including repetitive transcranial magnetic stimulation (rTMS) and transcranial direct current stimulation (tDCS), have demonstrated benefits in PTSD treatment (73–76). Whereas tDCS involves introducing low-intensity electric currents through the skull, rTMS involves repeated and rapidly changing electric currents on the surface of the skull. Both rTMS and tDCS have been shown to modulate cortical excitability in the brain (76, 77), and can improve PTSD symptoms, either as a monotherapy or as a treatment enhancement strategy with minimal side effects (73, 74, 78). However, data relating to treatment effects in PTSD are limited, and studies to clarify the exact mechanisms of action of tDCS and rTMS are warranted (75, 78).

Current clinical guidelines recommend treatment with serotonergic antidepressants; sertraline, fluoxetine, venlafaxine, or paroxetine as monotherapy have shown the greatest benefit with 58% of SSRI-treated patients exhibiting symptom improvement across clinical trials compared with 35% of placebo-treated participants (1, 7). For many SSRI drugs, effects in the amygdala have been demonstrated (**Table 1**). However, only sertraline and paroxetine are currently approved by the FDA for PTSD. Other pharmacological treatments have also been investigated for treating sleep disturbances in PTSD and include adrenergic inhibiting agents (prazosin, doxazosin), clonidine, tricyclic antidepressants, serotonin and alpha-adrenoceptor antagonists (trazodone, nefazodone, mirtazapine, cyclobenzaprine), atypical antipsychotics, gamma-aminobutyric acid modulators (gabapentin, topiramate), and cyproheptadine (115, 116). Additionally, while combined pharmacotherapy and psychotherapy approaches are recommended, randomized controlled trials have rarely shown them to be more effective than monotherapy. Therefore, additional studies of novel medications for use as monotherapy or in combination with psychotherapy to manage PTSD symptoms are warranted. A summary of candidate treatments for PTSD, detailing evidence of efficacy from clinical trials, and effects on the amygdala are given in **Table 1**.

**Table 1:** A summary of candidate PTSD treatment effects in the amygdala^a^.

Category	Treatment	Development status for PTSD	Evidence from clinical studies	Effects in the amygdala
**Neuropeptide**	Oxytocin	Phase II trial in progress (NCT04228289)	Facilitates encoding and consolidation of salient trauma-related memories (79) RCT found that intranasal oxytocin administration early post trauma reduced subsequent PTSD symptom development in recently trauma-exposed patients with high acute PTSD symptoms (80)	↓ Left amygdala-anterior insula connectivity in women with PTSD (81) ↓ Left amygdala reactivity in patients with PTSD (men + women) (82) ↓ Centromedial amygdala fMRI responses to fear signals (83) ↑ Extra-amygdala connectivity between the centromedial amygdala and frontoparietal regions (83)
**Orexin receptor antagonist**	Daridorexant	Phase II trial in progress (NCT05422612)	Improved sleep outcomes and daily functioning in patients with insomnia disorder (84)	Not anticipated to affect the amygdala
**SSRI**	Fluoxetine^b^	Phase II trial in progress (NCT05422612)	Improved PTSD symptoms (85)	↑ Anandamide in the BLA in mice (86) ↓ Inhibitory transmission in BLA in mice (86) ↓ c-Fos expression in BLA in mice (87)
	Vilazadone^b^	Phase II trial in progress (NCT05422612)	RCT reported no effect of vilazodone (40 mg) on PTSD symptoms and comorbid depression (88)	-
	Paroxetine^b^	FDA approved	Improved PTSD symptoms (re-experiencing, avoidance/numbing, and hyperarousal), social and occupational impairment, and comorbid depression (89, 90)	↓ Amygdala volume – possible correlation (91)
	Sertraline^b^	FDA approved	Improved PTSD symptoms (92–94)	Reversed ↑ amygdala activity during emotional processing of faces in patients with MDD (95)
**SNRI**	Venlafaxine^b^	Phase IV clinical trials (NCT04961190)	Improved PTSD symptoms (96)	-
**TRPC4/5 inhibitor**	BI 1358894	Phase II clinical trial (NCT05103657)	-	↓ Activity in bilateral amygdala and left anterior insula in response to negative emotional faces (97)
**NMDA receptor antagonist**	Ketamine	Phase II/III trials (NCT00749203; NCT02397889; NCT04560660)	Improved PTSD symptoms (98–101); no effect on PTSD symptoms (102)	↓ Activity in amygdala during the processing of positive and negative emotional faces in patients with MDD (103)
**Stimulant**	MDMA	Phase II clinical trials (NCT04030169)	Attenuated Clinician-Administered PTSD Scale for DSM-5 score and SDS scores, and improved self-reported symptoms when given in conjunction with psychotherapy in PTSD (104, 105) ↑ BDNF levels in the brain	↑ BDNF expression in amygdala in mice (106)
**Psychedelic**	Psilocybin	Phase I clinical trials (NCT05562973)	↓ Anxiety and depression in patients with cancer-related psychological distress (107) ↓ Depression in patients with MDD (108)	↓ Amygdala response to emotional stimuli in healthy humans (109) ↑ Amygdala response to fearful faces in patients with MDD (110) ↓ Top-down amygdala-visual cortex connectivity in healthy humans (111)
**DBS**	Amygdala DBS	Phase I trials (NCT03416894) (NCT02091843) in progress	-	↓ CCK-4 stimulated activity in BLA in mice (112)
**CBT**		In clinical trials (NCT03019497)	Improved PTSD symptoms (113)	↑ Amygdala activity associated with poorer CBT outcomes in PTSD (114) Bilateral amygdala activation in response to fear correlated with course of subsequent PTSD symptom change

a Candidate PTSD treatments with no current evidence of effects in the amygdala have not been included.

b Therapies with FDA approval for treatment of major depressive disorder. ↓ indicates a decrease, ↑ indicates an increase.

BDNF, brain-derived neurotrophic factor; BLA, basolateral amygdala; CBT, cognitive behavioral therapy; DBS, deep brain stimulation; DSM-5, Diagnostic and Statistical Manual of Mental Disorders 5^th^ Edition; fMRI, functional magnetic resonance imaging; MDD, major depressive disorder; MDMA, 3,4-methylenedioxymethamphetamine; NMDA, N-methyl-D-aspartate; PTSD, post-traumatic stress disorder; RCT, randomized controlled trial; SDS, Sheehan Disability Scale; SNRI, serotonin and norepinephrine reuptake inhibitors; SSRI, selective serotonin reuptake inhibitors; TRPC, transient receptor potential canonical ion channel.

Experimental adjunct treatments such as 3,4-methylenedioxymethamphetamine (MDMA), ketamine and psychedelics, for example psilocybin, have also been proposed for treating PTSD (117, 118). Recent randomized controlled trials in patients with chronic PTSD demonstrated that ketamine, an antagonist of the glutamate N-methyl-D-aspartate receptor, administered intravenously as either single or six repeated infusions over a 2-week period, resulted in clinically/significant improvements in PTSD symptoms compared with midazolam (98, 99). The benefits of ketamine in patients with PTSD and comorbid chronic pain or major depressive disorder (MDD) have also been demonstrated with comparable improvement in both PTSD and co-morbid symptoms (100, 101). However, a more recent study reported no significant effect of repeated ketamine administration over 4 weeks on PTSD symptoms (102). Similarly, varied results of ketamine on anxiety-like behaviors have been reported in animal models (119–122). Nevertheless, the magnitude of the effect on PTSD symptoms across other clinical trials, and the fact that esketamine is already FDA-approved for treating depression, suggest that the potential for ketamine as an alternative to SSRI treatments, or administered alongside psychotherapy, warrants further investigation in PTSD (118, 123). MDMA-assisted psychotherapy is currently in FDA Phase III clinical trials and is considered to facilitate psychotherapy by decreasing amygdala activation during therapeutic sessions, allowing participants to revisit distressing memories in a state of emotional security. Nevertheless, MDMA treatment has risks such as serotonergic depletion that can induce temporary states of anhedonia, lethargy, irritability, depression, anxiety, altered pain thresholds, and sleep disturbance, particularly in females (72, 124). Psychedelics and MDMA induce profound changes in perception, mood, and experiences of time and/or space, necessitating therapists to remain with patients while processing the experience and until the psychomimetic effects subside. As access to these treatments may be unavailable and/or cost prohibitive, newer treatments without such side effects or therapeutic burden are urgently needed.

### 2.3 Emerging therapy

#### 2.3.1 BI compound

**Figure 1:**
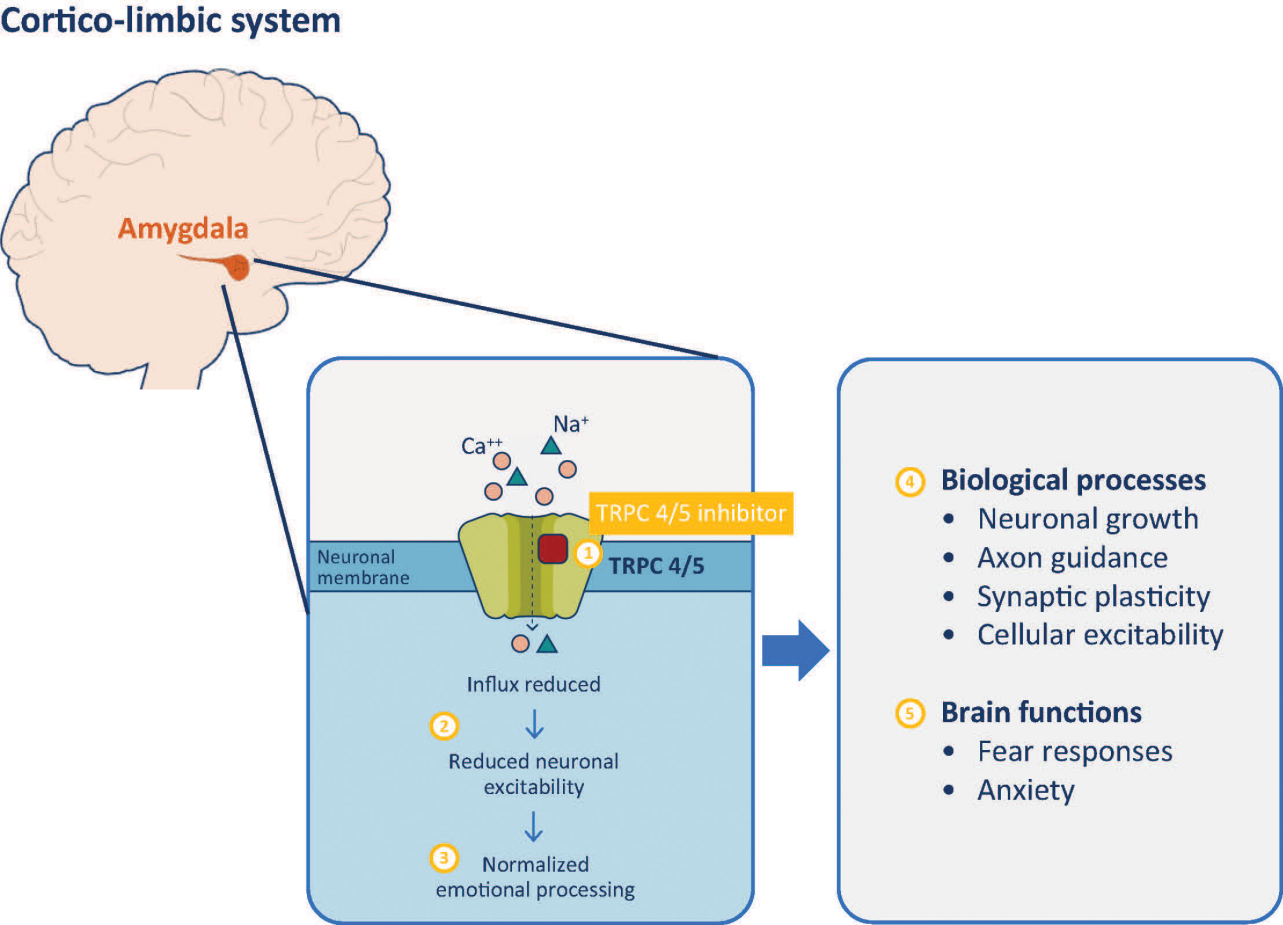
TRPC 4/5 inhibitor mechanism of action. Inhibition of TRPC 4/5 prevents influx of cations including calcium (1) and reduces neuronal activity of the amygdala (2). Reduced amygdala activity normalizes cortico-limbic system signaling, improving behaviors associated with dysfunctional emotional processing (3). These effects are mediated through modulation of biological processes (4) that impact on various brain functions of the amygdala (5). TRPC, transient receptor potential canonical ion channel.

In a Phase I clinical trial, BI 1358894 was well tolerated at doses of ≤200 mg, with a favorable pharmacokinetic (PK) profile in healthy Caucasian volunteers (125). Typically, TRPC4 and TRPC5 channels remain closed when not activated, which blocks calcium from entering the axon terminal, preventing the transmission of a neuronal signal and a physiological response (126, 127). When a stimulus is detected, TRPC4 and TRPC5 channels open, calcium enters the axon terminal and neuronal signals are transmitted, generating a physiological response (62, 126–128). Therefore, TRPC4/5 inhibition by BI 1358894 should reduce channel activation, preventing calcium influx, thereby dampening the related physiological responses (**Figure 1**).

Further study of BI 1358894 in 73 patients with MDD treated with BI 1358894 100 mg, citalopram 20 mg, or a matched placebo tablet, found reduced activation in several brain regions involved in emotional processing on fMRI, including the amygdala in those treated with BI 1358894 (97). BI 1358894 induced signal reduction in bilateral amygdala, the left anterior insula, the anterior cingulate cortex, and the left dorsolateral PFC in response to negative faces or scenes, whereas the attenuating effect of citalopram was limited to right amygdala activity in response to negative facial expressions (97). This agent may be beneficial in the reduction of PTSD symptoms. Given the association between the amygdala and PTSD, modulating the amygdala response with a pharmacologic agent could lead to improved compliance and completion of trauma-focused psychotherapy, thus providing greater PTSD symptomology improvements.

## 3 Discussion

Despite recent innovations in both psychological and pharmacologic interventions for PTSD, an unmet clinical need to improve treatment strategies remains (1). Given its significant prevalence and economic burden, novel treatment approaches are urgently needed to better manage symptoms.

## Author contributions

LD: Conceptualization, Writing – review & editing. MH: Conceptualization, Writing – review & editing.
